# Synthesis, Antibacterial Effects, and Toxicity of Licochalcone C

**DOI:** 10.3390/ph17050634

**Published:** 2024-05-14

**Authors:** Patrick Rômbola Ozanique, Alvaro Luiz Helena, Ralciane de Paula Menezes, Daniela Silva Gonçalves, Mariana Brentini Santiago, Guilherme Dilarri, Janaína de Cássia Orlandi Sardi, Henrique Ferreira, Carlos Henrique Gomes Martins, Luis Octávio Regasini

**Affiliations:** 1 Department of Chemistry and Environmental Sciences, Institute of Biosciences, Humanities and Exact Sciences, São Paulo State University (Unesp), São José do Rio Preto 15054-000, SP, Brazil; patrick.ozanique@unesp.br (P.R.O.); alvaro.helena@unesp.br (A.L.H.); 2 Department Microbiology, Institute of Biomedical Sciences, Federal University of Uberlândia (UFU), Umuarama 38405-320, MG, Brazil; ralciane@ufu.br (R.d.P.M.); dani_sg_1999@outlook.com (D.S.G.); mari.brentini@hotmail.com (M.B.S.); carlos.martins2@ufu.br (C.H.G.M.); 3 Department of Biochemistry and Microbiology, Institute of Biosciences, São Paulo State University (Unesp), Rio Claro 13506-900, SP, Brazil; gui_dila@hotmail.com (G.D.); henrique.ferreira@unesp.br (H.F.); 4 Dental Research Division, Guarulhos University, Guarulhos 07023-070, SP, Brazil; janasardi@gmail.com

**Keywords:** licochalcone, chalcone, *Glycyrrhiza*, membrane, biofilm, antibacterial, drug-resistant, *Galleria mellonella*

## Abstract

Drug-resistant bacteria constitute a big barrier against current pharmacotherapy. Efforts are urgent to discover antibacterial drugs with novel chemical and biological features. Our work aimed at the synthesis, evaluation of antibacterial effects, and toxicity of licochalcone C (LCC), a naturally occurring chalcone. The synthetic route included six steps, affording a 10% overall yield. LCC showed effects against Gram-positive bacteria (MIC = 6.2–50.0 µg/mL), *Mycobacterium* species (MIC = 36.2–125 µg/mL), and *Helicobacter pylori* (MIC = 25 µg/mL). LCC inhibited the biofilm formation of MSSA and MRSA, demonstrating MBIC_50_ values of 6.25 μg/mL for both strains. The investigations by fluorescence microscopy, using PI and SYTO9 as fluorophores, indicated that LCC was able to disrupt the *S. aureus* membrane, similarly to nisin. Systemic toxicity assays using *Galleria mellonella* larvae showed that LCC was not lethal at 100 µg/mL after 80 h treatment. These data suggest new uses for LCC as a compound with potential applications in antibacterial drug discovery and medical device coating.

## 1. Introduction

Antimicrobial resistance (AMR) is a challenge to human health, resulting in alarming numbers of deaths around the world. According to predictive statistical models performed by AMR collaborators, there was an estimate of 4.95 million (3.62–6.57) deaths associated with bacterial AMR in 2019 [1]. The discovery of new effective agents against multidrug-resistant bacteria is an urgent topic in global public health. The World Health Organization (WHO) indicated a human pathogen priority list, which included *Pseudomonas aeruginosa* (critical), *Staphylococcus aureus* and *Helicobacter pylori* (high), and *Streptococcus pneumoniae* (medium) [2]. Additionally, bacterial biofilms are barriers against current pharmacotherapy, which are constituted by cellular communities able to adhere to abiotic and biological surfaces, blocking the antibacterial drug penetration and diffusion into their complex extracellular matrix [3].

Natural products from plants are successful sources of antimicrobial drugs [4,5]. Out of 162 new antibacterial agents approved between 1981 and 2019, 55% are natural products and their derivatives, highlighting their relevance to modern drug discovery [6]. Among the promising bioactive natural products are retrochalcones, which are rare plant compounds and restricted to *Glycyrrhiza* genus. This taxon is constituted by several medicinal plants with evidenced antibacterial properties, including *Glycyrrhiza glabra*, popularly named licorice [7].

**Figure 1:**
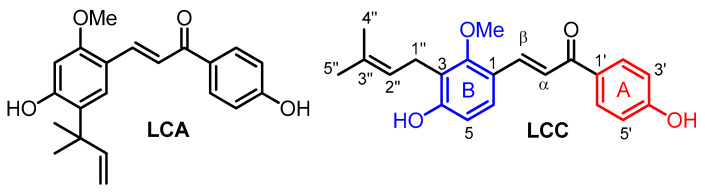
Structure of licochalcones A (LCA) and C (LCC).

Licochalcones are *C*-isoprenylated retrochalcones and have demonstrated remarkable antimicrobial activity [8]. Licochalcone A (LCA, Figure 1) is the most investigated retrochalcone and displayed a broad spectrum of effects against Gram-positive and Gram-negative species [9,10,11]. The effects of LCA on macromolecules of bacterial pathogens involved the inhibition of secretion of staphylococcal enterotoxins A and B, reduction of hemolysis by inhibiting the secretion, and production of staphylococcal α-toxin and inhibition of the release of suilysin by *Streptococcus suis* [12,13,14]. On the other hand, few biological studies of licochalcone C (LCC, Figure 1) were performed by former literature, which was strongly focused on its antitumor activity.

As part of our ongoing search for antibacterial drugs based on licochalcones from *G. glabra*, we first selected LCC due to its few synthesis, antibacterial, and toxicity studies in comparison to other licochalcones [15,16]. In this context, our aims were to provide evidence of the chemical and biological features of LCC as a rare plant active principle. In order to reach our perspectives, we synthesized LCC and tested its activity against a panel of Gram-positive, Gram-negative, and *Mycobacterium* species. In addition, studies on the effects of LCC against MSSA and MRSA biofilm formation and its mode of action on *S. aureus* membrane, as well as toxicity in *Galleria mellonella* larvae, were carried out.

## 2. Results and Discussion

### 2.1. Synthesis and Identification of Licochalcone C

**Scheme 1:**
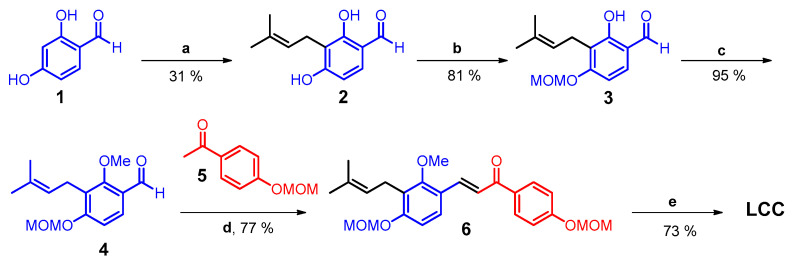
Synthetic route for LCC. (a) Isoprenyl bromide, NaOH, MeOH, rt, 12 h; (b) MOMCl, K_2_CO_3_, acetone, rt, 24 h; (c) CH_3_I, K_2_CO_3_, acetone, rt, 12 h; (d) NaOH, EtOH, H_2_O, rt, 36 h; (e) HCl, EtOH, THF, 70 °C, 6 h. MOM = methoxymethyl.

According to the literature data, few approaches to the synthesis of LCC have been reported to date. The LCC synthesis was described by Wang and collaborators, who achieved LCC (6% overall yield) and its regioisomer licochalcone H (LCH, 20% overall yield), using β-resorcylaldehyde (1) and 1,1-dimethylallyl alcohol as starting materials [17]. Kim and coauthors described the synthesis of LCC in six steps (7% overall yield), starting from **1** and isoprenyl bromide [18]. Dovhaniuk and colleagues developed a regioselective and scalable (up to 30 g) synthesis of LCC, using the direct *ortho*-metalation of resorcinol, allowing for the introduction of the isoprenyl group at C-3 [19]. Herein, we proposed a new route for the synthesis of LCC, which was based on previous approaches. We used a set of six steps, including non-regioselective *C*-isoprenylation at C-3 (step a), MOM-protection at O-4 (step b), *O*-methylation at O-2 (step c), Claisen–Schmidt condensation (step d), as well as MOM- deprotection of hydroxyl groups of rings A and B (step e), affording LCC with a 10% overall yield (Scheme 1).

The structure of LCC was confirmed by ^1^H and ^13^C nuclear magnetic resonance (NMR) and mass spectrometry (MS) spectral data analyses and comparison with former literature [20]. Two doublets (δ_H_ 7.99, 1H, *J* = 15.6 Hz for H-β; δ_H_ 7.68, 1H, *J* = 15.6 Hz for H-α) and δ_C_ 187.4 for carbonyl, δ_C_ 119.5 for C-α, and δ_C_ 138.4 for C-β corroborated an α,β-unsaturated ketone bridge, confirming the *trans*-chalconic structure of LCC. The ^1^H NMR spectrum exhibited two pairs of doublets at δ_H_ 8.04 (2H, *J* = 8.7 Hz, H-2′ and H-6′)/δ_H_ 6.96 (2H, *J* = 8.7 Hz, H-3′ and H-5′) and δ_H_ 7.65 (1H, *J* = 8.5 Hz, H-6)/δ_H_ 6.80 (1H, *J* = 8.5 Hz, H-5), which evidenced disubstituted ring A and tetrasubstituted ring B, respectively. Values of coupling constants of 8 Hz indicated three pairs of *ortho*-positioned hydrogens in both benzene rings. The methoxy substituent was confirmed by signals at δ_H_ 3.77 and δ_C_ 61.9. A set of signals in ^1^H NMR (δ_H_ 5.25, 1H, m; δ_H_ 3.37, 2H, d, *J* = 6.90 Hz; δ_H_ 1.78, 3H, s and δ_H_ 1.66, 3H, s) and ^13^C (δ_C_ 130.7, 123.0, 24.9, 22.7, and 17.1) confirmed the presence of the *C*-isoprenyl (or γ,γ-dimethylallyl) substituent. The MS spectrum in the positive mode exhibited a peak at *m*/*z* 339.17, which was attributed to the protonated molecule ion [M + 1]^+^, corroborating C_21_H_22_O_4_ as the molecular formula of LCC.

### 2.2. Antibacterial Assays

In order to assess the antibacterial activity of LCC, we tested its effects against seven Gram-positive, four Gram-negative, and three *Mycobacterium* species (Table 1).

LCC inhibited the growth of all Gram-positive bacteria, displaying minimum inhibitory concentration (MIC) values ranging from 6.2 μg/mL to 50.0 μg/mL. Among the Gram-positive strains, LCC was equally active against MSSA and MRSA (MIC = 12.5 µg/mL). Staphylococcal penicillin-binding proteins (PBP) are the β-lactam targets and MRSA strains can express PBP2a, an extra PBP with a low affinity to β-lactam, conferring resistance by staphylococci cells [21,22]. Thus, the identical susceptibility of MSSA and MRSA suggested LCC may not act in the set of PBPs. Our findings were corroborated by Wu and Avila, who identified the effects of LCC against clinical isolates of MSSA and MRSA [16,23]. Among Gram-negative species, *Helicobacter pylori* was susceptible to LCC (MIC = 25 μg/mL). LCC was inactive against *P. aeruginosa*, *K. pneumoniae,* and *Escherichia coli* (MIC > 400 µg/mL). The lack of effect against Gram-negative bacteria may be explained by their effective efflux pumps, which reduce the intracellular concentration of antibacterial compounds [24,25,26]. Kuete and collaborators reported that the association of an efflux pump inhibitor (EPI) with antibacterial drugs can restore or increase the effect of the antibiotics against Gram-negative bacteria. The efficacy of isobavachalcone (IBC), a *C*-isoprenylated chalcone, was associated with EPI. IBC was weakly active against Gram-negative strains when evaluated without EPI. On the other hand, the combination between IBC and EPI was able to reduce the MIC values, indicating that IBC was a substrate of efflux pumps in Gram-negative species, including *E. coli*, *P. aeruginosa*, *K. pneumoniae,* and *Enterobacter aerogenes* and *Enterobacter cloacae* [27]. In contrast to other bacterial pathogens, *H. pylori* has demonstrated a higher genetic heterogeneity due to the gastric adaptation. Its low clonality has led to genetic and phenotypical cells, which can be more susceptible than other tested Gram-negative species [28]. Finally, LCC exhibited antimycobacterial activity against *Mycobacterium tuberculosis*, *Mycobacterium avium,* and *Mycobacterium kansasii*, with MIC values ranging from 31.2 μg/mL to 125 μg/mL. Altogether, our data suggested that LCC had a narrow spectrum of antibacterial activity, focusing its effects against Gram-positive bacteria, *H. pylori,* and species of mycobacteria.

### 2.3. Checkerboard Assay

In antibacterial pharmacotherapy, the association of drugs is an effective tool to combat infections, enhancing the antibacterial potency as well as decreasing side effects, treatment period, and resistance [29]. Chalcones with diverse structural patterns have exhibited in vitro synergistic effects with antibacterial drugs. Gaur and co-authors described the anti-MRSA and synergistic effects of an indolyl-chalcone, which was able to reduce the MIC of norfloxacin by up to 16-fold and displayed fractional inhibitory concentration index (FICI) values < 0.5 [30]. Bozic and collaborators described the activity of hydroxychalcones against a panel of MRSA clinical isolates and their potent synergistic effect with norfloxacin, gentamicin, and trimethoprim-sulfamethoxazole [31]. LCC effects against MSSA (MIC = 12.5 µg/mL) and MRSA (MIC = 12.5 µg/mL) encouraged us to evaluate it combined with tetracycline (TC) and vancomycin (VAN). The checkerboard assay indicated FICI values > 0.5, demonstrating no synergistic effect between LCC and TC and VAN against MSSA and MRSA, respectively (Table 2).

### 2.4. Antibiofilm Assay

Biofilm is a central virulence factor of *S. aureus* pathogenesis and is responsible for recurrent and long-term staphylococcal infections. The high colonies-formation capacity of *Staphylococcus* species provides their adherence to biological and abiotic surfaces, including medical devices, establishing a suitable environment for bacterial survival [32]. In this context, biofilms act as a strong chemical and physical barrier against the penetration and diffusion of antibiotics, which should be used in higher concentrations to furnish satisfactory antibacterial action [33].

**Figure 2:**
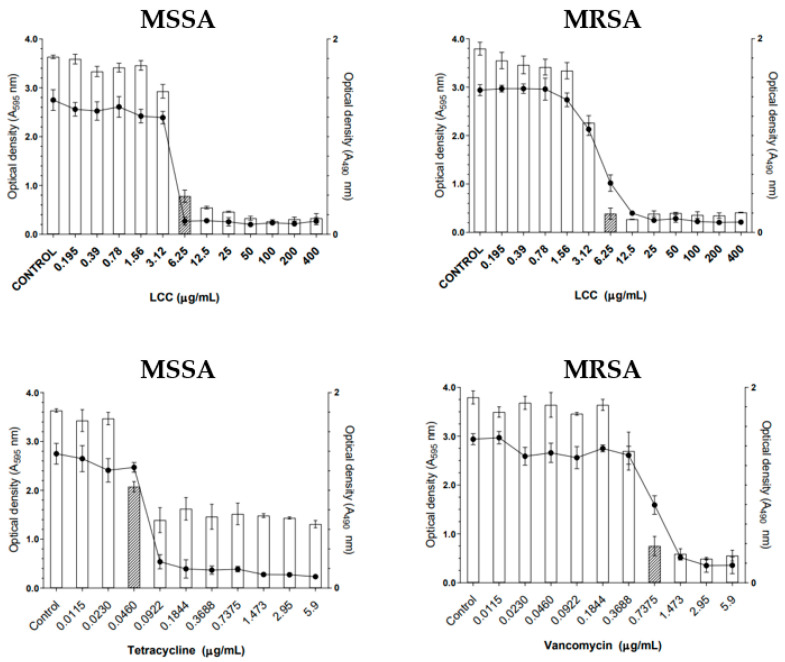
Effect of LCC (6.25 µg/mL) and the positive controls (tetracycline—0.046 µg/mL; vancomycin—0.7375 µg/mL) on MSSA and MRSA biofilm formation, respectively. The hatched bars represent the minimum biofilm inhibitory concentration (MBIC_50_) values. The lines represent the curve of the optical density (OD) values of biofilm metabolic activity in relation to the concentration of the tested sample.

The antibiofilm assay showed LCC was able to inhibit the biofilm formation of MSSA and MRSA strains in 75% and 87%, respectively, with MBIC_50_ values of 6.25 μg/mL. Tetracycline (MBIC_50_ = 0.046 µg/mL) and vancomycin (MBIC_50_ = 0.737 µg/mL) were used as positive controls and inhibited the biofilm formation of MSSA and MRSA, respectively (Figure 2).

### 2.5. Membrane Disruption Assay

Antibacterial drugs can act by well-established mechanisms of action, including the inhibition of metabolism (sulfonamides and trimethoprim), the inhibition of cell wall biosynthesis (β-lactams and glycopeptides), the disruption of protein biosynthesis (aminoglycosides, tetracyclines, amphenicols and macrolides, lincosamides, streptogramins, and oxazolidinones), and the inhibition of nucleic acid transcription and replication (quinolones and rifamycin) [34,35]. On the other hand, few antibacterial drugs have membranes as their target, highlighting the polymyxins. In this context, the development of antibacterial agents that act by membrane perturbation can be a possible way to avoid common and general resistance mechanisms [36].

**Figure 3:**
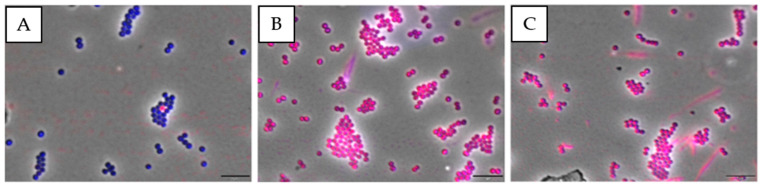
Fluorescence microscopy of *Staphylococcus aureus* stained with SYTO9 and propidium iodide. Cells stained blue and pink indicated whole and damaged membranes, respectively. (**A**) Negative control (cells treated with 1% DMSO); (**B**) positive control (cells treated with nisin); (**C**) cells treated with LCC at MIC value for 15 min. Magnification 100×; Scale bar 5 µm.

We evaluated the effects of LCC on the *S. aureus* membrane using fluorescence microscopy to evidence its antibacterial mode of action. Bacterial cells were treated with LCC at its MIC value (12.5 µg/mL) for 15 min, as well as nisin (positive control), an antibacterial peptide that targets the bacterial membrane, producing pores [37]. Two fluorescent dyes propidium iodide (PI) and SYTO9 were added, which stain cells with disrupted membranes (in pink) and whole membranes (in blue). Treatments with LCC and nisin exhibited pink cells and cells treated with 1% DMSO (negative control) demonstrated blue cells. Representative fluorescence images of the treatment of LCC, positive, and negative controls were presented in Figure 3.

**Figure 4:**
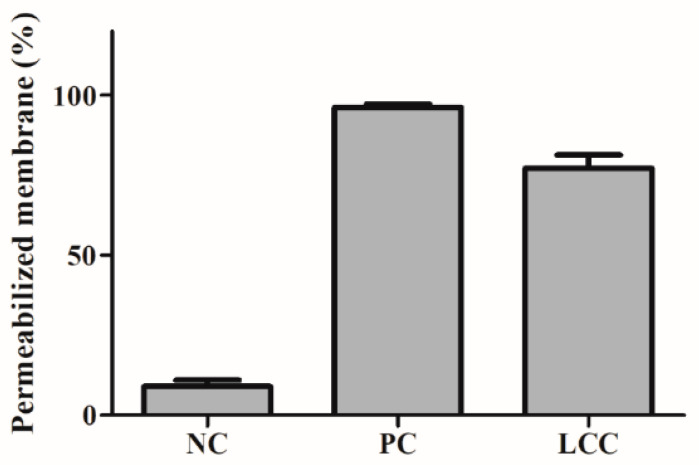
Percentage of *Staphylococcus aureus* cells with permeabilized membrane. NC (negative control): cells treated with 1% DMSO (negative control); PC (positive control): cells treated with nisin; LCC: cells treated with LCC (12.5 µg/mL).

The percentages of damaged cells with permeabilized membranes were quantified from the microscope images (Figure 4). Cultures treated with 1% DMSO had 10% of cells with permeabilized membranes. Treatments with nisin and LCC demonstrated 95% and 75% of the cells stained with both fluorophores, respectively, indicating their abilities to disrupt the *S. aureus* membrane.

Haraguchi and collaborators investigated the effects of LCC on the respiratory chains of *S. aureus* and *Micrococcus luteus*, indicating a blockage on membrane electron transport between coenzyme Q (CoQ) and cytochrome *c*. LCC was able to inhibit two bacterial membrane proteins NADH-oxidase (IC_50_ = 3.0 µM) and NADH-cytochrome *c* reductase (IC_50_ = 29.6 µM), and no effects on NADH-CoQ reductase (IC_50_ > 100 µM) and NADH-FMN oxidoreductase (IC_50_ > 100 µM). In addition, LCC did not inhibit the *E. coli* respiratory chain. Our findings corroborated the studies of Haraguchi and collaborators, considering that the membrane disruptor effects of LCC according to fluorescence microscope can directly cause conformational alterations in protein complexes responsible for the respiratory chain [15].

The inhibition of the bacterial respiratory chain and membrane disruptor activity may lead to some anxiety that LCC could affect eukaryotic electron transport and membranes, causing severe toxic effects. Thus, we evaluated the systemic toxicity of LCC on *Galleria mellonella* larvae.

### 2.6. Systemic Toxicity in Galleria Mellonella Larvae Assay

*G. mellonella* allows for the evaluation of the systemic toxicity of compounds due to some physiological similarities with mammalians [38,39]. In addition, *G. mellonella* is an invertebrate model that is more accepted by ethical committees than mice models. *G. mellonella* larvae techniques demand few resources and labor, due to their short life cycle (7–8 weeks), furnishing a rapid screening of toxicity [40].

**Figure 5:**
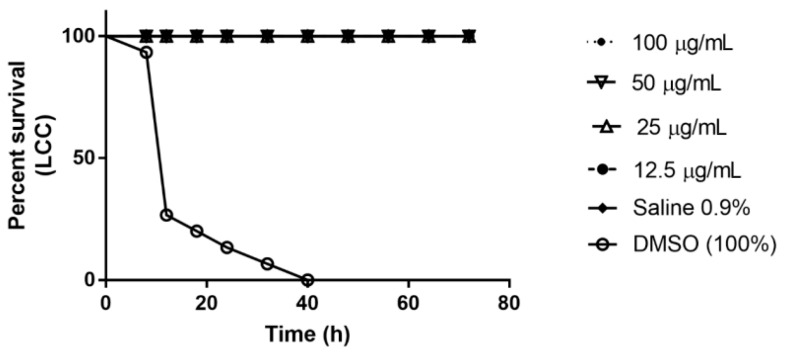
Systemic toxicity of LCC on *Galleria mellonella* larvae.

The larvae survival after treatments with vehicle control (saline 0.9%) and LCC at 12.5, 25, 50, and 100 µg/mL was 100% over a period of 72 h. On the other hand, the administration of 100% DMSO caused total lethality after 40 h (Figure 5).

Despite the non-toxic effects of LCC against *G. mellonella* larvae in the tested concentrations, some studies have reported its cytotoxic activity. LCC was able to inhibit the proliferation of human cancer cell lines, including T24 (bladder, 68% proliferation inhibition at 45 µg/mL), MCF7 (breast, 47% proliferation inhibition at 45 µg/mL), and A549 (lung, 40% proliferation inhibition at 45 µg/mL) [41], HCT116 (colorectal, IC_50_ = 16.6 µM) [42], HepG2 (liver, IC_50_ = 50.8 µM) [43], HN22 (oral, IC_50_ = 23.8 µM), HSC4 (oral, IC_50_ = 27.1 µM) [44], KYSE 30 (esophageal, IC_50_ = 28.0 µM), KYSE 70 (esophageal, IC_50_ = 36.0 µM), KYSE 410 (esophageal, IC_50_ = 19.0 µM), KYSE 450 (esophageal, IC_50_ = 28.0 µM), and KYSE 510 (esophageal, IC_50_ = 26.0 µM) [45]. In addition, LCC was also able to act against non-tumorigenic mammalian cells (Vero cells), demonstrating an IC_50_ of 27.7 µg/mL [16]. The cytotoxicity of LCC has been related to the regulation of some cellular signaling pathways, including JAK2/STAT3 and ROS/MAPK, as well as the activation of caspase-mediated cell death [44,45].

## 3. Materials and Methods

### 3.1. General Chemical Procedures

Reagents, solvents, and deuterated acetone were purchased from Merck^®^ (São Paulo, Brazil). Thin-layer chromatography (TLC) was performed on silica gel plates (8.0–12.0 µm, 200 µm, Supelco^®^_,_ St. Louis, MO, USA). Chromatography plates were visualized in a UV chamber at 254 nm and 365 nm and revealed by an anisaldehyde–sulfuric acid solution. Silica gel (230–400 mesh, Supelco^®^, St. Louis, MO, USA) and mixtures of hexane, acetone, and ethyl acetate were used for the chromatography column as stationary and mobile phases, respectively. ^1^H and ^13^C spectra were recorded on a Bruker^®^ Avance III spectrometer (14.1 T, 600 MHz) (Billerica, MA, USA). Low-resolution mass spectrometry by electrospray ionization spectra was performed on Bruker ^®^ Amazon ESI-IT (Billerica, MA, USA).

### 3.2. Synthesis of Licochalcone C

Synthesis of LCC was performed in six steps using well-established reactions in the former literature, including *C*-isoprenylation, MOM-protection, *O*-methylation, Claisen–Schmidt condensation, and MOM-deprotection.

#### 3.2.1. C-Isoprenylation Reaction of Compound **1** [46]

3,3-Dimethylallyl bromide (2.08 mL, 18 mmol) was slowly added to a stirred solution of β-resorcylaldehyde (1) (2.07 g, 15 mmol) and NaOH (0.6 g, 15 mmol) in methanol (20 mL) at 0 °C. The mixture was stirred for 12 h at room temperature and washed with water, extracted with ethyl acetate. The organic layer was dried with anhydrous magnesium sulfate and concentrated under reduced pressure. The crude product was purified over flash silica gel column chromatography (hexane:ethyl acetate = 10:1) to furnish 2 as a white solid in a 31% yield.

#### 3.2.2. Protection Reaction of Compound **2** [47]

Chloromethyl methyl ether (0.41 mL, 5.4 mmol) was slowly added to a solution of 2 (0.93 g, 4.5 mmol) and K_2_CO_3_ (1.86 g, 13.5 mmol) in dry acetone (15 mL) at 0 °C. The mixture was stirred for 24 h at room temperature, washed with water, and extracted with ethyl acetate. The organic layer was dried with anhydrous magnesium sulfate and concentrated under reduced pressure. The crude product was purified over silica gel column chromatography (hexane:ethyl acetate = 4:1) to afford 3 as a colorless oil in an 81% yield.

#### 3.2.3. O-Methylation of Compound **3** [48]

Iodomethane (0.34 mL, 5.25 mmol) was added to a solution of 3 (0.87 g, 3.5 mmol) and K_2_CO_3_ (1.93 g, 14 mmol) in dry acetone (15 mL) at 0 °C. The mixture was stirred for 12 h at room temperature, washed with water, and extracted with ethyl acetate. The organic layer was dried with magnesium sulfate and concentrated under reduced pressure. The crude product was purified over silica gel column chromatography (hexane:ethyl acetate = 4:1) to afford 4 as a yellow oil in a 95% yield.

#### 3.2.4. Synthesis of Compound **5** [47]

Chloromethyl methyl ether (0.67 mL, 8.76 mmol) was slowly added to a solution of 4′-hydroxyacetophenone (1.0 g, 7.3 mmol) and K_2_CO_3_ (3.03 g, 21.9 mmol) in dry acetone (15 mL) at 0 °C. The mixture was stirred for 8 h at room temperature, washed with water, and extracted with ethyl acetate. The organic layer was dried with anhydrous magnesium sulfate and concentrated under reduced pressure. The crude product was purified over silica gel column chromatography (hexane:ethyl acetate = 4:1) to afford 5 as a colorless oil in a 75% yield.

#### 3.2.5. Claisen–Schmidt Condensation Reaction of Compounds **4** and **5** [49]

An aqueous solution of NaOH (60% *w*/*v*, 6.4 mL) was slowly added to a solution of 4 (0.84 g, 3.2 mmol) and compound **5** (0.63 g, 3.5 mmol) in ethanol (16 mL) at 0 °C. The mixture was stirred for 36 h at room temperature, washed with water, and extracted with ethyl acetate. The organic layer was dried with magnesium sulfate and concentrated under reduced pressure. The crude product was purified over flash silica gel column chromatography (hexane:ethyl acetate:acetone = 7:2:1) to afford 5 as a yellow oil in a 77% yield.

#### 3.2.6. Deprotection Reaction of Compound **6** [49]

An aqueous solution of hydrochloric acid (1 mol/L, 10 mL) was slowly added to a solution of 6 (0.98 g, 2.3 mmol) in ethanol (10 mL) and THF (10 mL) at 0 °C. The mixture was stirred for 6 h at 70 °C, washed with water, and extracted with ethyl acetate. The organic layer was dried with magnesium sulfate and concentrated under reduced pressure. The crude product was purified over flash silica gel column chromatography (hexane:ethyl acetate:acetone:dichloromethane = 6:2:1:1) to afford LCC as a yellow oil in a 73% yield.

### 3.3. Identification of Licochalcone C

The structure of LCC was identified by ^1^H NMR, ^13^C NMR, and MS spectral data analyses. All NMR parameters, including hydrogen and carbon chemical shifts (δ_H_ and δ_C_), integrations, multiplicities (s = singlet; d = doublet and m = multiplet), and coupling constants (*J* in Hz), corresponded to the structure, and were compared to former literature reports [20].

^1^H NMR (600 MHz, acetone-*d*_6_): δ_H_ 8.04 (d, *J* = 8.7 Hz, 1H, H-2′ and H-6′), 7.99 (d, *J* = 15.6 Hz, 1H, H-β), 7.68 (d, *J* = 15.6 Hz, 1H, H-α), 7.65 (d, *J* = 8.7 Hz, 1H, H-6), 6.96 (d, *J* = 8.7 Hz, 1H, H-3′ and H-5′), 6.80 (d, *J* = 8.5 Hz, 1H, H-5), 5.28–5.23 (m, 1H, H-2″), 3.77 (s, 3H, OCH_3_), 3.37 (d, *J* = 6.9 Hz, 2H, H-1″), 1.78 (s, 3H, H-5″), 1.66 (s, 3H, H-4″).

^13^C NMR (150 MHz, acetone-*d*_6_): δ_C_ 187.4 (C=O), 161.6 (C-4′), 159.7 (C-4), 158.8 (C-2), 138.4 (C-β), 130.8 (C-2′ and C-6′), 130.7 (*C*-3″), 130.6 (C-1′), 126.5 (C-6), 123.0 (C-2″), 122.1 (C-3), 120.2 (C-1), 119.5 (C-α), 115.2 (C-3′ and C-5′), 111.9 (C-5), 61.9 (OCH_3_), 24.9 (C-1″), 22.7 (C-5″), 17.1 (C-4″).

MS (positive mode, electrospray, Mwt.: 338.40): *m*/*z* = 339.17 [M + 1]^+^. NMR and MS spectra of LCC are presented in the Supplementary Material.

### 3.4. Antibacterial Assays

The strains were purchased from the American Type Culture Collection (ATCC) (Manassas, VA, USA) and were maintained in the culture collection of the Laboratory of Antimicrobial Testing in the Federal University of Uberlandia, from Minas Gerais, Brazil.

The antibacterial and antimycobacterial effects of LCC were tested against MSSA (ATCC 6538), MRSA (ATCC BAA44), *S. epidermidis* (ATCC 14990), *E. faecalis* (ATCC 1299), *S. pneumoniae* (ATCC 6305), *S. sanguinis* (ATCC 10556), *S. sobrinus* (ATCC 33478), *S. mutans* (ATCC 25175), *H. pylori* (ATCC 43526), *P. aeruginosa* (ATCC 27853), *K. pneumoniae* (ATCC 23883), *Escherichia coli* (ATCC 25922), *M. tuberculosis* (ATCC 27294), *M. avium* (ATCC 25291), and *M. kansasii* (ATCC 12478).

The MIC values against Gram-positive and Gram-negative species were determined in triplicate using the broth microdilution method in 96-well microplates as previously reported, using resazurin as a colorimetric indicator of cell viability [50]. LCC was dissolved in dimethyl sulfoxide (DMSO) at 1 mg/mL, followed by dilution in brain heart infusion (BHI) to achieve the final concentrations ranging from 400 to 0.195 μg/mL. The final DMSO content was 5% (*v*/*v*) and a similar solution was used as a negative control. The inoculum was adjusted for each microorganism to reach a cell suspension of 5.0 × 10^5^ colony-forming units per mL (CFU/mL), as preconized by the Clinical and Laboratory Standards Institute (CLSI), with modifications in the culture medium [51]. The growth control (inoculated well) and sterility control (non-inoculated well free of antimicrobial agent) were evaluated. Tetracycline, vancomycin, and chlorhexidine were used as positive controls and evaluated in independent assays in concentrations ranging from 0.0115 μg/mL to 5.9 μg/mL, 0.0115 μg/mL to 5.9 μg/mL, and 0.115 μg/mL to 59 μg/mL, respectively. The 96-well microplates were sealed with plastic film and incubated at 37 °C for 24 h. After this period, 30 μL of aqueous solution of resazurin (0.02%) was added to the microplates and incubated for 15 min at 37 °C. The MIC value was defined as the lowest concentration able to inhibit the microorganism growth (color change of resazurin from blue to pink), which was expressed in μg/mL. The assay was conducted in triplicate.

The MIC values of LCC against mycobacteria species were determined according to the protocol of Palomino and collaborators, with slight modifications [52,53]. A stock solution of LCC was prepared in DMSO and diluted in Middlebrook 7H9 broth to achieve the final concentrations ranging from 7.8 μg/mL to 1000 μg/mL. Isoniazid was dissolved in DMSO and used as a positive control in concentrations ranging from 0.015 μg/mL to 1 μg/mL. The inoculum was prepared by introducing a range of colonies grown in Ogawa–Kudoh in a tube containing glass beads with 500 μL of sterile water. An aliquot of 200 μL was transferred to a tub containing 2 mL of 7H9 broth, incubated at 37 °C for seven days, and compared with McFarland scale 1 (3.0 × 10^8^ cells/mL). The inoculum was suspended in 96-well plates at a 1:25 ratio with 7H9 broth. The growth controls (without antibiotics) and sterility controls (without inoculation) were also included. The 96-well plates were incubated at 37 °C for seven days. After this period, 30 μL of an aqueous solution of resazurin 0.02% was added to each well, which was incubated for 24 h at 37 °C. The MIC value was defined as the lowest compound concentration able to inhibit the mycobacterial growth, which was expressed in μg/mL. The assay was conducted in triplicate.

### 3.5. Checkerboard Assay

The combinations of LCC with tetracycline and vancomycin were evaluated against MSSA and MRSA, respectively, using a microdilution broth checkerboard assay, according to White and collaborators, adapted with the standard procedure established by the CLSI [51,54]. The fractional inhibitory concentration (FIC) of the combination between LCC and tetracycline or vancomycin was determined using Mueller–Hinton broth culture medium into 96-well plates, with a final inoculum suspension of 5.0 × 10^5^ CFU/mL, incubated at 37 °C for 24 h. After incubation, an aqueous resazurin solution (0.02%) was added to the wells and the FIC values (ratio between the MIC of the drug in combination and the MIC of the drug alone) were determined for each drug (Equations (1) and (2)). The fractional inhibitory concentration index (FICI) was calculated using Equation (3) [30].

(1) $${FIC}_{LCC}=\frac{MIC\;of\;drug\;A\;in\;combination}{MIC\;of\;drug\;A\;alone}$$

(2) $${FIC}_{D}=\frac{MIC\;of\;drug\;B\;in\;combination}{MIC\;of\;drug\;B\;alone}$$

FIC Index (FICI) = FIC_LCC_ + FIC_D_ (3)

The combination was classified as synergistic (FICI ≤ 0.5), indifferent (0.5 < FICI < 4), and antagonistic (FICI ≥ 4) [54]. The assays were performed in triplicate in independent experiments.

### 3.6. Antibiofilm Assay

The LCC inhibition against the biofilm formation of MSSA and MRSA was evaluated using the broth microdilution methodology proposed by the CLSI (2012) [51]. The minimum biofilm inhibitory concentration (MBIC_50_) was established as the concentration of LCC able to inhibit 50% or more of biofilm formation [55]. The MBIC_50_ values were determined using two protocols, including biomass and cell viability assessment by optical density (OD) reading. Two microplates were used for each protocol. The assays were performed in triplicate in three independent experiments. The inoculum concentration and optimal incubation time for this assay were determined by standardizing biofilm formation. In 96-well flat-bottom microplates containing BHI broth supplemented with 2% glucose, serial dilutions of the samples were made from the stock solution (1600 µg/mL), obtaining a final concentration between 0.195 µg/mL and 400 µg/mL. From a 24 h culture on BHI agar plates, the inoculum of MSSA and MRSA was prepared in BHI broth supplemented with 2% glucose, with equivalent turbidity on a spectrophotometer at 625 nm, to match 0.5 in the McFarland scale (1.5 × 10^8^ CFU/mL). The bacterial suspensions were diluted to the final concentration of 1.0 × 10^6^ CFU/mL. The microplates were incubated at 37 °C for 24 h. Subsequently, the contents of the wells were aspirated, and non-adhered cells were removed by washing with a phosphate-buffered saline (PBS) buffer (pH = 7.2). The formed biofilm was fixed with methanol for 15 min, dried at room temperature, and stained with a crystal violet solution (0.2%) for 20 min. After removing the crystal and washing the wells with the PBS buffer, 33% acetic acid was added to solubilize the crystal retained in the biofilm. The absorbance of the wells was determined in a spectrophotometer at 595 nm. The determination of MBIC_50_ was performed using Equation (4) [55,56].

MBIC_50_ = 1 − (A_595_ of the test ÷ A_595_ of non-treated control) × 100 (4)

The evaluation of the ability of the samples to inhibit or reduce the viability of the biofilm formed was performed as proposed by Parai et al. (2020) and Saising et al. (2012), with modifications. Briefly, the plates intended for this evaluation were prepared in the same way as those reserved for biomass evaluation by the crystal violet method and incubated for 24 h. Then, the entire contents of the wells were gently aspirated, and the wells were washed with PBS to remove non-adherent cells. Subsequently, 50 μL of the menadione solution and 2,3-bis(2-methoxy-4-nitro-5-sulfophenyl)-2H-tetrazolium-5-carboxanilide (MTT) were added with a final concentration of 0.5 mg/mL and the plates incubated in the dark for 2 h at 37 °C. Then, formazan crystals (product of MTT reduction by metabolically active cells present in the biofilm) were solubilized by adding 100 μL of dimethyl-sulfoxide (DMSO) for 10 min, and 80 μL of each well were transferred to another plate, wherein reading was performed in a spectrophotometer with a wavelength of 490 nm [57,58].

Tetracycline and vancomycin were used as positive controls and were evaluated at concentrations ranging from 0.0115 µg/mL to 5.9 µg/mL. Bacterial cells were evaluated in the absence of the antibacterial compounds and were used as negative controls.

### 3.7. Membrane Disruption Assay

The assay was performed using *Staphylococcus aureus* (ATCC 29213), an MSSA strain. Bacterial cells were cultivated in solid TSA broth (tryptone soya) at 36 °C and stirred at 200 rpm. *S. aureus* (10^6^ cells) was exposed to LCC at its MIC value (12.5 µg/mL) in DMSO for 15 min in a 1.5 mL microcentrifuge tube in a total volume of 100 μL of TSA medium. Next, 900 μL of the TSA medium was added to the tube to dilute the compound and stop the reaction [59]. Cells were stained with the Live/Dead BacLight Kit (Thermo-Scientific^®^ L7012, Carlsbad, CA, USA), which is composed of two nucleic acid dyes: SYTO9, which stains all cells, and propidium iodide, which penetrates cells with damaged membranes [60]. The stained cells were immobilized on agarose-covered slides as described by Martins [61]. Untreated *S. aureus* cells with intact membranes were used as negative controls, and *S. aureus* cells exposed to nisin (Sigma-Aldrich^®^ N5764, St. Louis, MO, USA) were used as positive controls for membrane damage [62]. Microscopic analyses were carried out using an Olympus BX-61 microscope, equipped with a monochromatic OrcaFlash-2.8 camera (Hamamatsu, Japan), and the software CellSens Dimension Version 11 (Olympus).

### 3.8. Systemic Toxicity in Galleria Mellonella Larvae Assay

The systemic toxicity assay was performed to evaluate the acute toxic effects of LCC, following the procedures described by Megaw [63]. For the experiment, 10 healthy larvae from *G. mellonella* were randomly selected for each concentration test, with a weight range between 0.2 g and 0.3 g and without melanization aspects. The larvae were chilled on ice for 20 min and had their prolegs cleaned with 70% ethanol. Four solutions of LCC in DMSO were prepared with different concentrations, ranging from 25 µg/mL to 100 µg/mL. Five microliters of the solution were injected into the hemocoel of each larva through the last left proleg using a 25 µL Hamilton syringe. After the administration, the larvae were incubated at 37 °C and their survival was monitored in selected intervals over 72 h. The larvae unable to move when touched and showing elevated levels of melanization were counted as dead. Experiments were performed in triplicate.

## 4. Conclusions

In summary, we synthesized and evaluated LCC as part of our ongoing search for antibacterial agents. LCC demonstrated effects against Gram-positive bacteria, *H. pylori,* and *Mycobacterium* species. The combination of LCC-tetracycline and LCC-vancomycin exhibited no synergistic effects against MSSA and MRSA. LCC was able to inhibit MSSA and MRSA biofilm formation and its mode of action involved *S. aureus* membrane disruption. Furthermore, systemic toxicity investigation using *G. mellonella* larvae indicated LCC was not toxic. Altogether, our findings open new ways for LCC as an antibacterial agent, with potential applications in drug discovery and medical device coatings.

## Figures and Tables

**Table 1:** Antibacterial activity of LCC expressed as MIC values (in µg/mL) *.

Species			MIC (μg/mL)			
		LCC	Tetracycline ^#^	Vancomycin ^#^	Chlorhexidine ^#^	Isoniazid ^#^
Gram-positive	*Staphylococcus aureus* (MSSA)	12.5	0.09	-	-	-
	*Staphylococcus aureus* (MRSA)	12.5	-	0.73	-	-
	*Staphylococcus epidermidis*	6.2	>5.9	-	-	-
	*Enterococcus faecalis*	50.0	3.3	-	-	-
	*Streptococcus pneumoniae*	50.0	0.4	-	-	-
	*Streptococcus sanguinis*	6.2	-	-	1.8	-
	*Streptococcus sobrinus*	12.5	-	-	3.6	-
	*Streptococcus mutans*	12.5	-	-	0.4	-
Mycobacteria	*Mycobacterium tuberculosis*	31.2	-	-	-	0.5
	*Mycobacterium avium*	62.5	-	-	-	>1.0
	*Mycobacterium kansasii*	125.0	-	-	-	1.0
Gram-negative	*Helicobacter pylori*	25.0	1.47	-	-	-
	*Pseudomonas aeruginosa*	>400	5.9	-	-	-
	*Klebsiella pneumoniae*	>400	1.47	-	-	-
	*Escherichia coli*	>400	0.73	-	-	-

* MIC values were the mean of triplicates. MSSA: methicillin-susceptible *Staphylococcus aureus*; MRSA: methicillin-resistant *Staphylococcus aureus*. -: not tested; ^#^ positive controls.

**Table 2:** Effect of the combination of LCC with antibacterial drugs against MSSA and MRSA planktonic cells.

	MIC (μg/mL)								
*S. aureus* Strains	Combination	Alone		Combined		FIC_LCC_	FIC_D_	FICI	Type of Combination
		LCC	D	LCC	D				
MSSA	LCC + TC	12.5	0.09	3.12	0.18	0.25	2.0	2.25	Indifferent
MRSA	LCC + VAN	12.5	0.73	6.25	0.36	0.5	0.5	1.0	Indifferent

TC: tetracycline; VAN: vancomycin; D: drug-associated (TC or VAN) to LCC against the *S. aureus* strain; FIC_LCC_: fractional inhibitory concentration for LCC (Equation (1)); FIC_D_: fractional inhibitory concentration for the associated drug (Equation (2)); FICI: fractional inhibitory concentration index (Equation (3)).

## Data Availability

Data are contained within the article and Supplementary Materials.

## Supplementary Materials

The following supporting information can be downloaded at: https://www.mdpi.com/article/10.3390/ph17050634/s1, NMR and MS spectra. Figure S1: ^1^H NMR spectrum of LCC (acetone-*d*_6_; 600 MHz); Figure S2: Amplification of ^1^H spectrum of LCC (1.5–8.0 ppm) (acetone-*d*_6_; 600 MHz); Figure S3: Amplification of ^1^H spectrum of LCC (6.75–8.05 ppm) (acetone-*d*_6_; 600 MHz); Figure S4: Amplification of ^1^H spectrum of LCC (5.1–5.4 ppm) (acetone-*d*_6_; 600 MHz); Figure S5: Amplification of ^1^H NMR spectrum of LCC (3.34–3.78 ppm) (acetone-*d*_6_; 600 MHz); Figure S6. Amplification of ^1^H NMR spectrum of LCC (1.8–1.6 ppm) (acetone-*d*_6_; 600 MHz); Figure S7: ^13^C NMR spectrum of LCC (acetone-*d*_6_; 150 MHz); Figure S8: MS spectrum of LCC performed by electrospray (positive mode).
