# Supplementary material for: Synthesis, Antibacterial Effects, and Toxicity of Licochalcone C
Source: Pharmaceuticals (Basel). 2024 May 14;17(5):634. doi: 10.3390/ph17050634 (PMC11124413; doi:10.3390/ph17050634)
Supplement: Supplementary file 1 [file pharmaceuticals-17-00634-s001.zip › pharmaceuticals-2966281-supplementary.pdf]

Synthesis, Antibacterial Effects, and Toxicity of Licochalcone C

SUPPLEMENTARY MATERIAL

Patrick Rômbola Ozanique [1], Alvaro Luiz Helena [1], Ralciane de Paula Menezes [2], Daniela Silva Gonçalves [2], Mariana Brentini Santiago [2], Guilherme Dilarri [3], Janaína de Cássia Orlandi Sardi [4], Henrique Ferreira [3], Carlos Henrique Gomes Martins [2] and Luis Octávio Regasini [1,*]

[1] Department of Chemistry and Environmental Sciences, Institute of Biosciences, Humanities and Exact Sciences, São Paulo State University (Unesp), São José do Rio Preto 15054-000, SP, Brazil; patrick.ozanique@unesp.br (P.R.O.); alvaro.helena@unesp.br (A.L.H.)

[2] Department Microbiology, Institute of Biomedical Sciences, Federal University of Uberlândia (UFU), Umuarama 38405-320, MG, Brazil; ralciane@ufu.br (R.d.P.M.); dani_sg_1999@outlook.com (D.S.G.); mari.brentini@hotmail.com (M.B.S.); carlos.martins2@ufu.br (C.H.G.M.)

[3] Department of Biochemistry and Microbiology, Institute of Biosciences, São Paulo State University (Unesp), Rio Claro 130506-900, SP, Brazil; gui_dila@hotmail.com (G.D.); henrique.ferreira@unesp.br (H.F.)

[4] Dental Research Division, Guarulhos University, Guarulhos 07023-070, SP, Brazil; janasardi@gmail.com

* Correspondence: luis.regasini@unesp.br; Tel.: +55-17-32212362

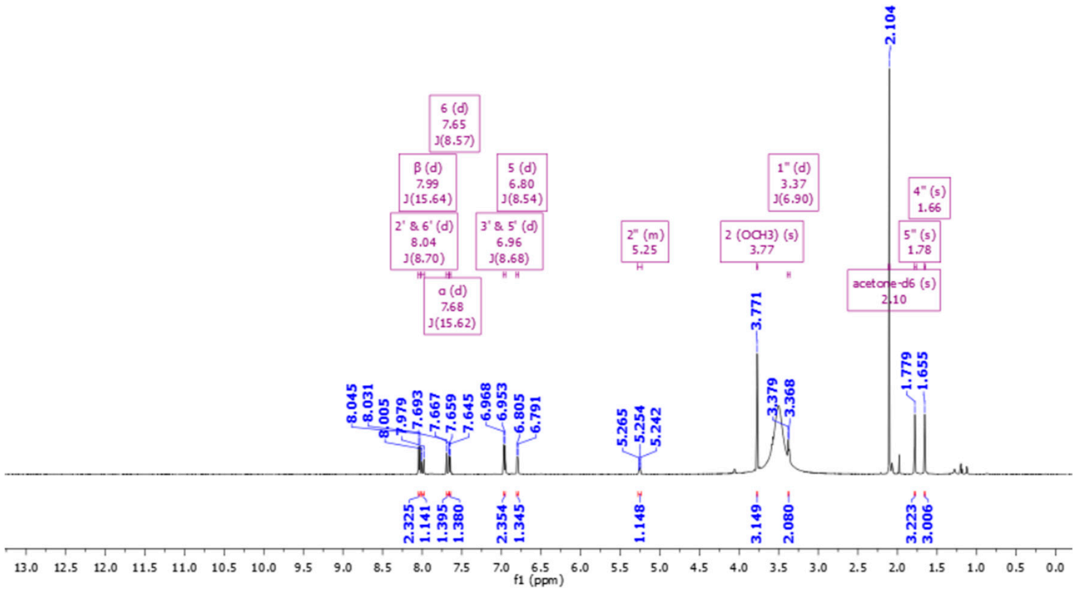

Figure S1. ^{1}H NMR spectrum of LCC (acetone-d_6; 600 MHz)

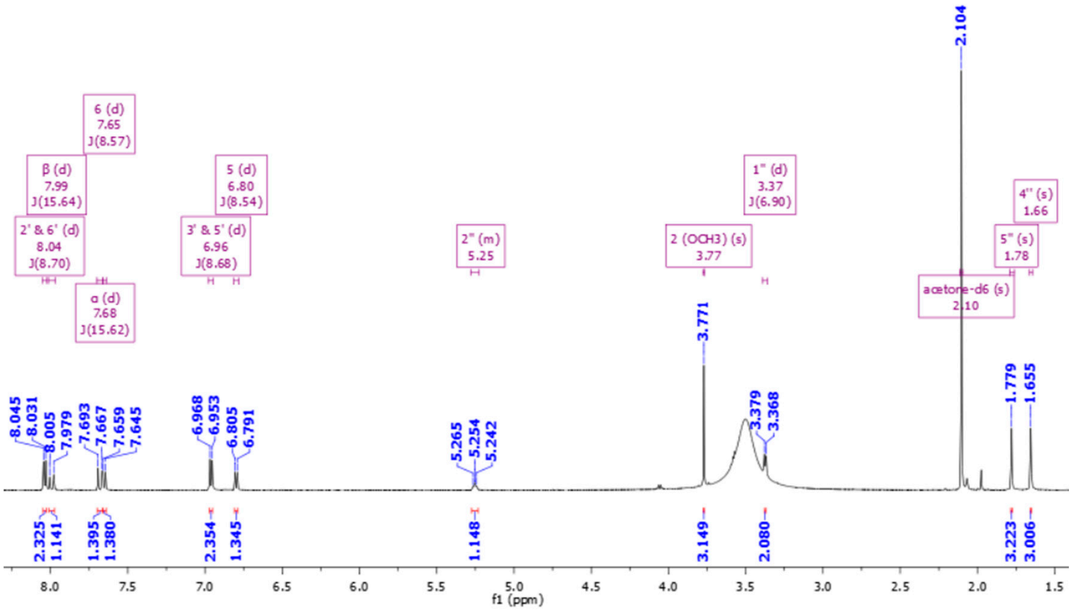

Figure S2. Amplification of ^{1}H spectrum of LCC (1.5 – 8.0 ppm) (acetone-d_6; 600 MHz)

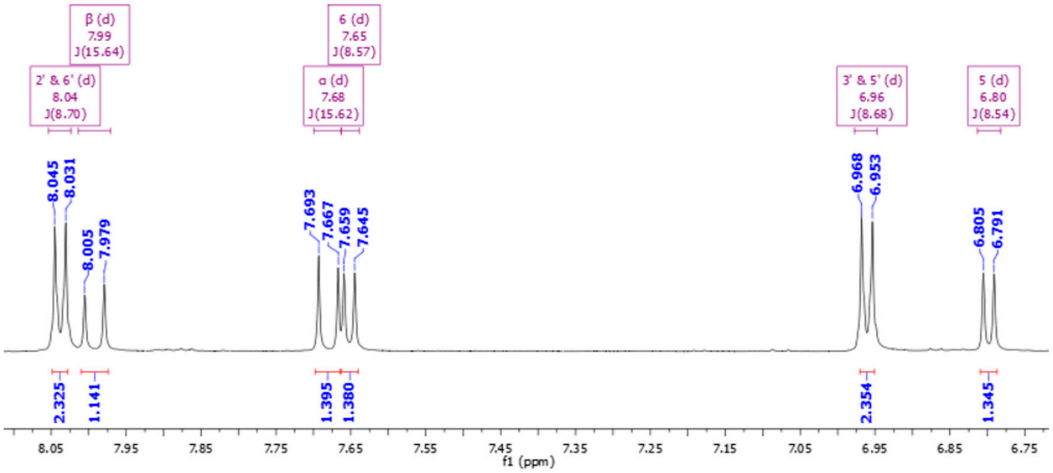

Figure S3. Amplification of ^{1}H spectrum of LCC (6.75 – 8.05 ppm) (acetone-d_6; 600 MHz)

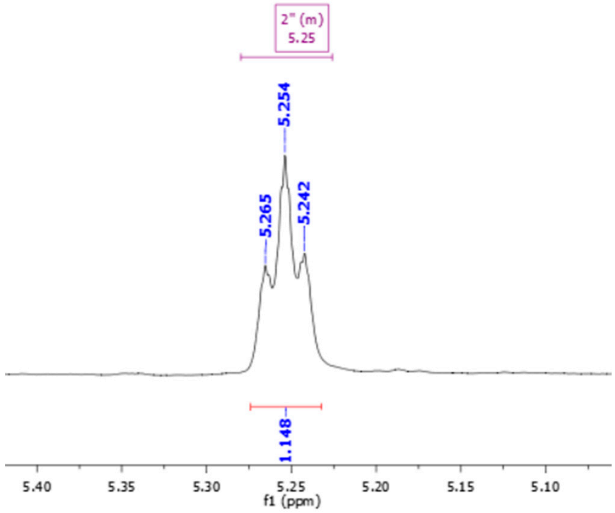

Figure S4. Amplification of ^{1}H spectrum of LCC (5.1 – 5.4 ppm) (acetone-d_6; 600 MHz)

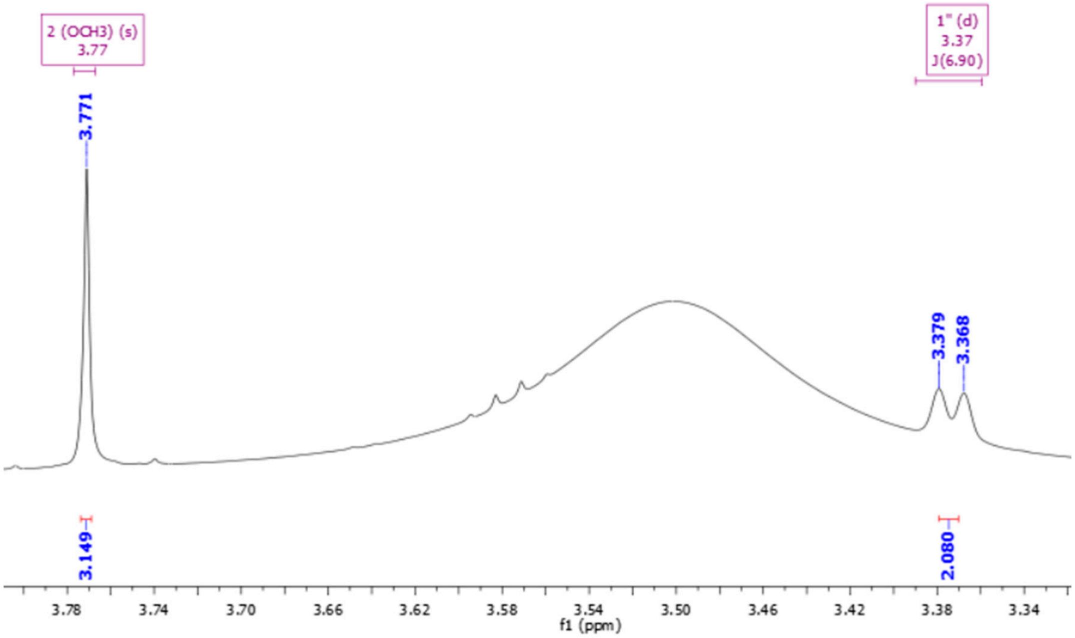

Figure S5. Amplification of ^{1}H NMR spectrum of LCC (3.34 – 3.78 ppm) (acetone-d_6; 600 MHz)

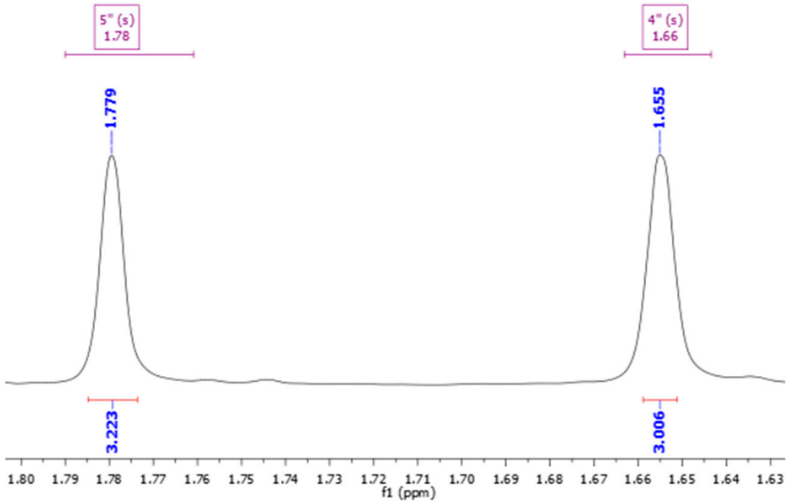

Figure S6. Amplification of ^{1}H NMR spectrum of LCC (1.8 – 1.6 ppm) (acetone-d_6; 600 MHz)

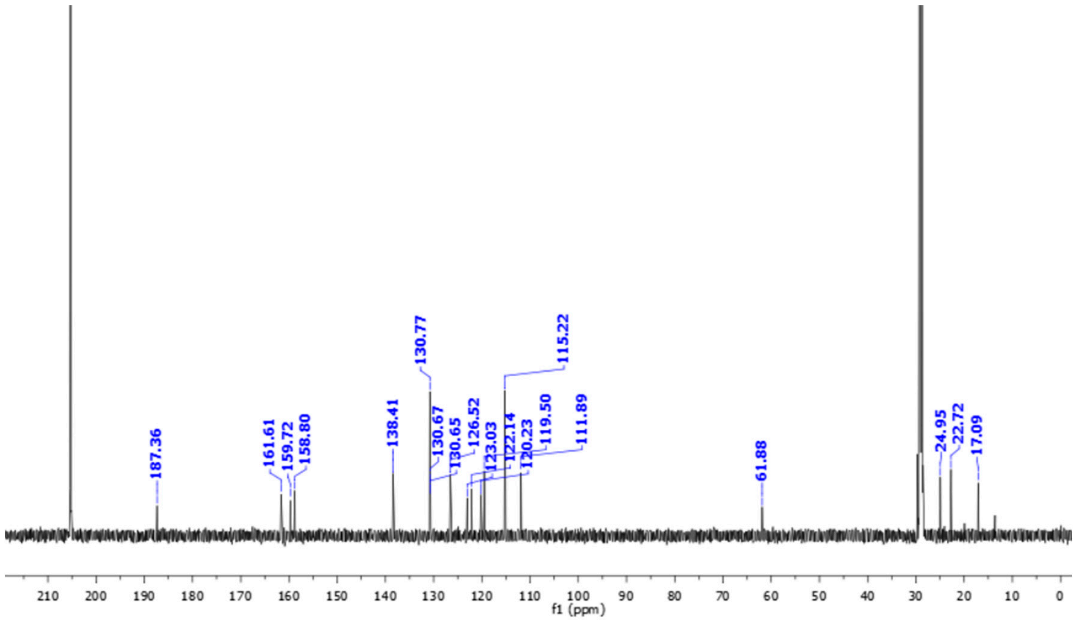

Figure S7. ^{13}C NMR spectrum of LCC (acetone-d_6; 150 MHz)

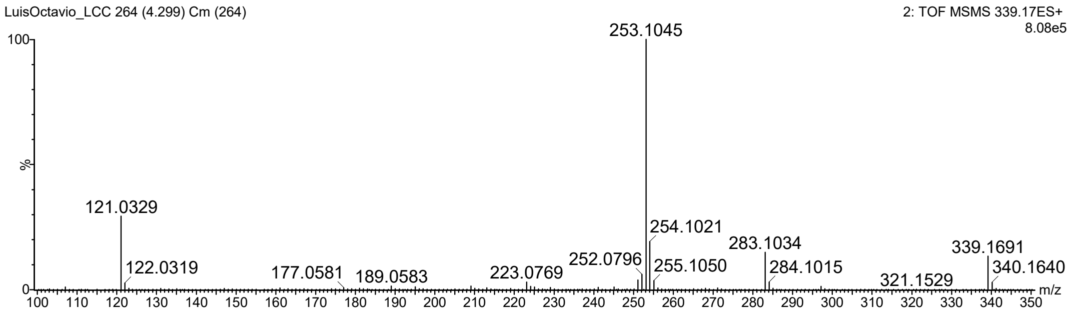

Figure S8. MS spectrum of **LCC** performed by electrospray (positive mode).
